# Factors associated with acute kidney injury and outcomes in patients with malaria in a district hospital in Rwanda

**DOI:** 10.4314/ahs.v24i3.12

**Published:** 2024-09

**Authors:** Larrisa Umuhire, Violette Dushimiyimana, Michel Nkuranyabahizi, Flavien Ngendahayo, Jean Claude Shyaka, Innocent Ngerageze, Lakshmi Rajeswaran, Geldine Chironda

**Affiliations:** 1 University of Rwanda, College of Medicine and Health Sciences, School of Nursing and Midwifery, Kigali, Rwanda; 2 Seed Global Health, Sant John of God College of Health Sciences, Mzuzu, Malawi

**Keywords:** Acute kidney injury, severe malaria, complications of malaria

## Abstract

**Introduction:**

Acute kidney injury (AKI) remains one of the complications of severe malaria. Evidence on associated factors and outcomes for patients with complicated malaria and AKI is limited in Rwanda.

**Aim:**

To assess the factors associated with acute kidney injury and outcomes in patients with malaria in a district hospital in Rwanda.

**Method:**

A retrospective study design was applied. A census sampling strategy was used to select 122 files of patients admitted as severe malaria patients in 2016-2017. A developed clinical audit form was used to collect data from patients' files. Both descriptive and inferential statistics were used to analyze data.

**Results:**

Among the confirmed severe malaria files, 44% of participants were over 50 years and 52.5% were males. The majority, (91.5%) had community-based health insurance and 16.3% had acute kidney injury. The significant associated clinical factors were dehydration (p=.01), high-grade fever (p=.002), profuse sweating (p=.034), vomiting (p=.043), and diarrhea (p=.025). Of the 20 patients who developed AKI, 55% completely recovered, 15% died and 30% of cases were transferred to the highest facilities for hemodialysis.

**Conclusion:**

The existence of AKI among severe malaria patients was evident with some recovering and others dying. There is a need for educating healthcare professionals, mostly at district hospitals about the diagnosis and management of AKI as a result of complicated malaria.

## Introduction

Malaria is an infectious mosquito-borne and most prevalent endemic disease affecting millions of people living mostly in poor tropical and subtropical areas of the world^1^. In many of the countries affected, it is a leading cause of illness and death^2^. According to Boushab et al., 3 and Engels et al.^4^ Malaria is one of the top 10 killer diseases in the world. In 2019, there were an estimated 409,000 global malarial deaths, which rose to 627,000 deaths in 2020^4^,^5^. Kidney complications in malaria mainly occur due to hemodynamic dysfunction and immune response^6^,^7^,^8^. Liver complications leading to hepatomegaly, jaundice, and hepatic dysfunction can also contribute to the occurrence of acute kidney injury (AKI)^9^,^10^.

The WHO Afro-region carries a disproportionately high share of the global malaria burden^2^,^11^. Moreover, AKI because of malaria in this region is evident^12^. Kidney Disease Improving Global Outcomes (KIDGO)^13^ defined AKI as a sudden loss of kidney function based on increased serum creatinine levels (a marker of kidney excretory function), reduced urinary output (a quantitative marker of urine production) and is limited to a duration of 7 days. Olowu and colleagues reveals 66% of children and 70% of adults in need dialysis due to AKI. Although the authors reported malaria as the cause of AKI in children and adults, the outcomes were not indicated. Moreover, the same authors highlight a mortality rate of 34% in children and 32% in adults, which further rose to 73% in children and 86% in adults when dialysis is not received^14^.

In Rwanda, there has been an increase in the annual incidence of malaria (cases per 1000 population at risk) from 203.44 in 2015 to 404.88 in 2016 with a proportional rise in severe malaria from 12.092 in 2015 to 17.248 in 2016. Additionally, the total number of annual deaths of malaria (death per 100 000 population at risk) increased from 514 in 2015 to 715 in 2016^15^. Among these cases, AKI is an associated complication as identified by Igiraneza et al^16^. Similarly, a study done by Odiit et al^17^ reveals AKI in 6.8% of children with sepsis and critical illness and a mortality rate of 36.2%. Severe malaria is associated with significant multi-organ dysfunction including AKI, further long hospitalization and mortality^18^,^19^. Additionally, detrimental effects of long-term behavioral problems, long-term neurocognitive impairment, and chronic kidney disease with severe malaria^20^,^21^,^22^ are causes for concern. Evidence of the existence of AKI in severe malaria patients has been identified in Rwanda^16^,^17^,^23^. In the context of the current study, the prevalence of malaria is higher in Rwanda with the Eastern province recording 17%, compared to southern province (11%), Kigali city (3%), Western Province (2%) and North province (1%). Nyamata District Hospital is found in Bugesera district within the Eastern Province. The prevalence of malaria in the Bugesera Districts is 12.71%^24^,^25^. However, the information on the associated factors and outcomes for patients with complicated malaria and AKI is limited. Therefore, this current study aimed at assessing the associated clinical factors and outcomes of AKI in patients with severe malaria at a selected district health setting in Rwanda.

## Methodology

### Study design and setting

A quantitative approach was utilized. A retrospective cross sectional design was applied to assess factors associated with AKI and outcomes in severe malaria patients at Nyamata district hospital in Rwanda. The study setting is one of the 36 district hospitals in Rwanda and the main referral from the 15 primary level health centres and one prison dispensary located in its catchment area^26^. The hospital currently receives 80% from Bugesera District and 20% patients from other nearby districts thus serving approximately 18,789 individuals in the province^26^. To service the population, the district hospital contains 18 departments and scope of services includes inpatient and outpatient diagnosis as well as treatment^26^.

The clinical services provided include curative and rehabilitative services, Support preventive and promotional activities within the catchment area, high dependency, accident and emergency services, dental services, and operating theatres^27^. Out of the approximate 182 staff members, 6 laboratory technicians, 12 are general practitioners, 13 midwives and 62 are registered nurses^28^. Nurses as part of the multidisciplinary team are involved in treatment of severe malarial patients at Nyamata district hospital. At primary health care centres, nurses are involved in consulting, diagnosing and treatment of uncomplicated malarial and referral of complicated malarial cases to district hospitals. Hence the consideration of this hospital for the current study.

### Study Population, sample, and sampling

Files of patients admitted with severe malaria from 2016 to 2017 were included. Excluded from the study were files with incomplete information. According to the health information system (HIMS) from 2016 to 2017, severe malaria hospitalized cases were 176. However, 122 files were included based on containing full information. A census sampling strategy was used as all files who met the inclusion criteria were considered for the study.

### Data collection instrument

The clinical audit form for the current study was developed from in-depth literature 29,30. The form was composed of three parts with a total of 22 items. Part one collected data on demographic characteristics, including information on confirmed diagnosis of malaria. Part two assessed the patient's clinical characteristics (8 items): dehydration, hypovolemia, high-grade fever, profuse sweating, vomiting, diarrhea, and jaundice. The last part collected information on the outcomes of patients with severe malaria using the following: recovery, transfer to central hospitals for further management, and death.

AKI is defined as the rise in serum creatinine ≥0.3 mg/dL within 48 hours, serum creatinine ≥ 1.5 times baseline, which is known or presumed to have occurred within the prior seven days and urine output < 0.5 mL/kg/hour for six hours. This definition is based on the widely accepted Kidney Disease: Improving Global Outcome (KDIGO) definition of AK1^31^.

### Validity and reliability

The tool of this study was developed by a researcher based on in-depth literature from different studies thus enhancing content validity. The tool was given to two experts from the clinical (nephrologists and general practitioners) and academic areas (nephrology nursing lectures) who verified the content. Alignment of the specific objectives with the sections of the checklist was done to make sure that all the content was covered. Furthermore, the researcher selected 15 patients' files and gave them to rater one to check and rate it against the tool being tested. Then, the same files were given to rater two to do follow the same procedure done by rater one. The two raters agreed on all the items. The internal consisitency (Cronbach alpha) was 0.73, meaning the tool was a good measure of variables under study.

### Data collection procedure

After obtaining the ethical clearance, the researcher approached, and introduced herself to the director of nursing for permission to access the patient's files. The second day after being allowed to access the information, the researcher selected all patients with severe malaria as confirmed by the diagnosis directly from the case files from January 2016 to December 2017. After finding severe malaria cases, the researcher chose all the files of a sample to be checked. During coding, the first code corresponded to the first admission patient file in January 2016 and the last code would be the last patient file admitted in December 2017. After coding the patient's files, data collection commenced. The data was collected during daily working hours from 8 am to 5 pm for 14 days

### Data analysis

Data were cleaned, coded and entered into the Statistical package for social sciences (SPSS) version 21.0 in preparation for analysis. Descriptive statistics were used to present demographic data and factors related to and outcomes of patients with a confirmed diagnosis of AKI. Inferential statistic test of chi-square was used to establish the clinical factors associated with AKI among malaria patients. P value set at less than or equal to .05.

### Ethical considerations

Before collecting data, the permission was obtained from the Institutional Review Board of the University of Rwanda College of Medicine and Health Sciences (CMHS/IRB/082/2019). In addition, the Director General of Nyamata District Hospital provided a written permission to conduct the study. Confidentiality of collected information was assured by not putting any identification information from the patient's file on the clinical audit form. In addition, the collected data were kept in a locked cupboard for hard copies. Data in SPSS was stored in a passworded computer.

## Results

### Social demographic characteristics

Approximately 44% of patients were over 40 years of age, with more males (52.5%) than females. About 62% of patients had not attended school and 72.1% depended on agriculture as their occupation. The majority (91.5%) had community-based health insurance.

### Proportion of severa malaria patients with AKI

Out of the 122 files cases selected with severe malaria, 16.3% had confirmed diagnosis of AKI while the majority (80.3%) did not have. There were only 3.3% file cases with no documentation.

### Clinical Factors associated with acute kidney injury

Among cases who developed AKI, vomiting was a major factor to contribute to AKI (65%%), followed by high grade fever (55%), dehydration(45%) and profuse sweating (40%). Whilst for non AKI cases, only 39% had highgrade fever, followed by vomiting (25%) and profuse sweating (20%). The associated clinical factors were dehydration (p=.001), hypovoleamia (p=.005), high-grade fever (p=.20), profuse sweating (p=.05), vomiting (p=.001), and diarrhea (p=.005).

### The outcome of acute kidney injury

According to table 3, the outcomes of the AKI cases were recovery (55%), 30% transferred for dialysis and 15% died.

**Table 3:** Proportion of symptoms/signs in AKI vs non-AKI patients

Nature of symptom	AKI patients (n=20)		Non-AKI patients (n=102)		Chi square	P value
	Yes	No	Yes	No		
Dehydration	9 (45%)	11 (55%)	10 (9%)	92 (91%)	15,75	0.001*
Hypovolemia	4 (20%)	16 (80%)	3 (3%)	99 (97%)	9,00	0,005
High-grade fever	11 (55%)	9(45%)	40 (39%)	62 (61%)	1,71	0,20
Profuse sweating	8 (40%)	12 (60%)	20 (20%)	82 (80%)	3,93	0.05*
Vomiting	13 (65%)	7 (35%)	25 (25%)	77 (75%)	12,78	0.001*
Diarrhoea	6 (30%)	14 (70%)	8 (8%)	94 (92%)	8,08	0,005

**P value set at less than or equal to .05**

## Discussion

The study revealed all age groups, from one year, mostly women and farmers to be affected by severe malaria in Rwanda thus confirming the findings by Izere et al.^32^, Ndahiro et al.^33^, and Hakizayezu et al.^34^. However, a study was done in Tanzania and Angola indicated more males (65.4% & 59% respectively) being affected by malarial infection^19^,^35^. The majority of the study sample had community-based health insurance (CBHI) and it is the only insurance that assists the majority of the population in Rwanda^36^. Among 122 reviewed files, approximately 16% were diagnosed with AKI thus almost similar to studies done in India and Tanzania where 19.9% and 18.3% of patients with severe *P. falciparum* malaria were diagnosed with AKI respectively^35^. Compared to the current findings, some studies revealed even a higher figure of 27.18% and 31.7% of severe malaria cases having AKI^37^,^38^,^39^. The evidence is likely to vary to some degree, may be depending on multiple factors like missed diagnosis, better prevention and earlier treatment.

Although limited knowledge of CKD does not necessary impact AKI, this was identified by Igiraneza and colleagues among healthcare professionals^40^. This finding might also translate to underdiagnosis of AKI with other patients even dying from the disease before confirmation. Similarly, the limited knowledge in management of CKD in healthcare professionals in Rwanda^40^,^41^ might make effective management of AKI is impossible. While effective implementation of interventions on malarial treatment in different districts of Rwanda^23^,^38^,^42^ might be the other reason for lower prevalence of AKI, the population continues to suffer the complication of severe malaria.

Patients diagnosed with AKI presented with clinical features such as high-grade fever, vomiting, profuse sweating, dehydration, diarrhea, and hypovolemia as revealed by the findings of the current study. These results are supported by Akobye^43^, Kute, et al.^44^, Karoli et al.^45^, and Shashidar^46^. It is interesting to note that the symptoms reported are those of malaria and may be associated with risk of AKI, but are not symptoms of AKI per se. Therefore, healthcare professionals should monitor urine output and further screening for AKI through frequent renal function tests should also take precedence in scenarios like these.

A study done by Sacomboio et al.^19^ indicated blood pressure and increased temperatures 37.2 degrees or more as other clinical risk factors of AKI although fever was not an associated factor in the current study. In low-resource areas like rural and districts healthcare settings of Rwanda where there is limited laboratory diagnostics^47^, vital signs like temperature and blood pressure may be used as an indicator of kidney damage as highlighted by Sacomboio et al.^19^. On the contrary, the findings of Saravu et al.^48^ indicated malaria fever as a non-associated feature of patients with AKI. While the current study revealed oliguria, abdominal pain/tenderness, and jaundice as clinical symptoms of AKI, tachycardia, hepatomegaly, splenomegaly and hyper parasitemia, severe anemia, hemoglobinuria, repeated seizures, clinical jaundice, cerebral malaria, and shock are also found among severe malaria patients^43^,^45^,^46^.

The identified clinical symptoms of dehydration, hypovolemia, profuse sweating, vomiting and diarrhoea are the prerenal causes of kidney failure. They result in depletion of the intravascular volume and blood pressure, with further reduction of blood flow to the kidneys, consequently decrease in kidney function as indicated by Batte et al.^49^ and Wandile^50^. Therefore, fluid resucitation in such circumstances is critical, however, clinicians should avoid fluid overload to to avoid precipitating the need for dialysis and death. It is important for clinicians to monitor andeasure kidney function in severe malaria patients as they are a risk group for AKI. Failure will result in missed opportunities to treat the AKI thus leading to CKD that requires more aggressive therapies like dialysis^49^ and in worst case scenarios, death^51^. Moreover, proper treatment of the underlying cause (malaria) is encouraged to prevent the malarial infection induced hypercatabolic state which increases the need for diaysis^52^,^53^. However, in situations of AKI complications, immediate referral for dialysis to higher level care institutions is required to save life^54^.

Out of the population with confirmed AKI, 55% had a good recovery, 15% died and 30% were transferred to higher care facilities for heamodialysis. Recovery from AKI was indicated by attainment of normal values of serum creatinine and urine output criteria (per KDIGO) within 7 days after AKI onset as indicated by Chawla et al.^55^. Patients were transferred to higher care facilities for heamodialysis after presenting with pulmonary oedema, hyperkaleamia, uremia and uremia encephalopathy as outlined by Igiraneza et al.^16^ Prasad and Mishra^56^ indicated a higher death percentage of 25.8 of patients who were in acute kidney failure stage. A retrospective cohort study in Sudan reveals complete renal recovery in 35.7% of patients and 31.2% died^57^. A study by Thanachartwet et al.^58^ indicated the requirement for RRT in 45.2% with in-hospital mortality of 31.9% among patients with malaria diagnosed with AKI. Therefore, proper management of malaria compounded with AKI reduces the need for costly dialysis in majority^59^ and promotes recovery of the affected population.

## Limitations of the study

The study was carried out at one of the district hospitals, hence the findings cannot be generalized to all the district healthcare centers in Rwanda. Difficulties in interpreting information found in the documents due to jargon and acronyms as well as non-verification of the information were the limitations. Moreover, a prospective study should be considered since the current design is considered inferior to the latter. Although it is possible to evaluate other malaria related complications such as anaemia, hemoglobinuria and seizures using the history of the patient at district hospital level, these were not captured in the notes. Although 176 files were eligible for the current study, 122 case files qualified as some of them had missing information. This significant missing information from the eliminated files poses information bias.

## Conclusion

The prevalence of aki in malaria patients especially in severe cases was evident though low compared to other studies. Whilst severe malaria is not a common diagnosis among adults in sub-Saharan Africa, the current study reveals many adult patients with severe malaria. High-grade fever, vomiting, profuse sweating, dehydration, diarrhea, and hypovolemia were the contributing factors of AKI in malaria patients. More than half of the patients appropriately recovered from malaria-related AKI, but another significant portion of cases were transferred for dialysis services while others died. Although the prevention and management of malaria are crucial, there is a need to sensitize healthcare professionals, mostly nurses at district hospitals about the severity of AKI which comes as a result of complicated malaria. Another recommendation is for the available nephrology trained nurses to make scheduled visits to healthcare centers to aid in assessing complicated malarial patients for the signs and symptoms of AKI for further management.

## Figures and Tables

**Table 1:** Socio-demographic data (n = 122)

Variables	Frequency(n)	Percentage (%)
**Age (years)**		
1-4	1	0.8
10-19	10	8.2
20-39	26	21.3
30-49	31	25.4
40-49	16	13.1
50 plus	38	31.1
**Gender**		
Male	63	52.5
Female	59	47.5
**Level of education**		
Primary school	9	7.4
Secondary school	30	24.6
Higher school	7	5.7
None	76	62.3
**Occupation**		
Agriculture	88	72.1
Employee	6	4.9
Private	4	3.3
Student	7	5.7
No documentation	17	13.9
**Medical insurance of participants**		
CBI	111	91.0
Security boards	10	8.2
None	1	0.8

**Table 2:** Proportion of severe malaria patients with AKI (n=122)

Variable	Frequency	Percentage
Confirmed diagnosis of AKI		
Yes	20	16.4
No	98	80.3
No documentation	4	3.3

**Table 4:** The outcome of severe malaria patients with AKI (n=20)

Variables	AKI & Malaria
Recovery	11(55%)
Transferred for further management	6 (30%)
Death	3 (15%)
**Total**	**20 (100%)**
